# Computed tomography features and clinicopathological characteristics of gastric glomus tumor

**DOI:** 10.1186/s12876-022-02241-w

**Published:** 2022-04-09

**Authors:** Jing-jing Xing, Wen-peng Huang, Fang Wang, Ya-ru Chai, Jian-bo Gao

**Affiliations:** Department of Radiology, The First Affiliated Hospital of Zhengzhou University, No. 1, East Jianshe Road, Zhengzhou, 450052 Henan Province China

**Keywords:** Glomus tumor, Gastric, Tomography, X-ray computed, Differential diagnosis

## Abstract

**Background:**

Gastric glomus tumor (GGT) is a rare neoplasm that is difficult to distinguish from other gastric submucosal tumors due to a lack of diagnostic experience. The goal of this study was to better understand GGT by looking at its clinicopathological features, computed tomography (CT) features, and differential diagnosis.

**Methods:**

The clinical data and CT findings of 21 pathologically confirmed GGT patients were examined. The clinical characteristics and CT findings of benign GGT were compared to gastric stromal tumors (GST) (n = 30) and heterotopic pancreas (n = 30).

**Results:**

The 21 cases included six males and fifteen females ranging in age from 42 to 64 years. The lesions were found in the gastric body in four cases and the antrum in seventeen. GGT was diagnosed as benign in 20 cases and malignant in one. In benign cases, the glomus cells were small, uniform, showed perivascular hemangiopericytoma‑like or solid nest‑like structures. Obvious mitotic figures were observed in the malignant case. SMA staining was positive in the tumor cells. A quasi-round or round solid mass protruded into the gastric cavity in 20 benign cases, with a clear and smooth edge. The long to short diameter ratio was 1.01 ± 0.15. All of the benign cases had obvious enhancement, with homogeneous enhancement in ten cases and heterogeneous enhancement in ten cases, as well as central filling enhancement in 12 cases. The ratio of CT value of lesion to abdominal aorta in arterial phase and venous phase were (0.41 ± 0.11) and (0.81 ± 0.20), which were significantly higher than GST and heterotopic pancreas. The irregular mass broke through the gastric wall and invaded liver with poorly defined boundary and internal necrosis, heterogeneous persistent moderate enhancement with thickening blood supply arteries was seen in one malignant case with a long diameter of 150 mm and a thick diameter of 30 mm.

**Conclusions:**

CT enhancement usually shows persistent obvious enhancement, especially in arterial phase, which provides important value for the diagnosis. CT findings can help in the differential diagnosis of GGT and other submucosal tumors.

## Background

Glomus tumors (GTs) are rare benign mesenchymal neoplasms originating from the glomus of the anastomoses of small arteries, usually occurring in the peripheral soft tissues, especially in the distal parts of fingertips or toes [1]. Glomus tumors rarely occur in the stomach [2]. GGT is typically reported as case report [3–8]. As lack of diagnostic experience in imaging features, it is difficult to distinguish it from other gastric submucosal tumors perioperatively. In the present study, a total of 21 cases of GGTs confirmed by pathology were collected and analyzed. The clinicopathological features and CT findings were compared with those of gastric submucosal tumors such as gastric stromal tumor, heterotopic pancreas, with the goal of improving GGT diagnosis and narrowing the differential diagnosis.

## Materials and methods

### Patients

This is a retrospective study that was approved by the Ethics Committee of Zhengzhou University’s First Affiliated Hospital, which waived the requirement for written informed consent due to the patients’ anonymity. Between January 2008 and June 2021, patients who met the following criteria were collected retrospectively in our hospital. Inclusion criteria: ① GGT confirmed by postoperative pathology; ② Abdominal CT plain scan and contrast-enhanced scan were performed preoperatively. The clinical and pathological data were complete. Exclusion criteria: ① There were obvious artifacts on the image; ② The lesion was too small to be recognized. All patients’ clinical manifestations and tumor marker results were documented.

### CT evaluation

A total of fifteen cases were examined using the GE Discovery 750 HD CT and six using the Toshiba 320 CT. The scanning parameters for the former machine were: tube voltage of 120 kV, automatic mA, pitch of 0.984, thickness of 5 mm; and the scanning parameters for the latter machine were: tube voltage of 120 kV, tube current of 350 mA, pitch of 0.813, scan thickness and the increment of 5 mm. After an intravenous injection of nonionic iohexol (iopromide, 370 mg/mL, GE Medical Systems, 1.5 mL/kg and 3 mL/s) through a dual-head pump injector (Medrad, Warrendale, PA, United States), contrast-enhanced images of the arterial phase and portal venous phase of each patient were acquired at 25 s and 60 s respectively.

According to the gender, age (± 3 years) and diagnosis year of each GGT patient matched with GST and heterotopic pancreas patients, 30 GST and heterotopic pancreas patients were retrieved from picture archiving and communication system of our hospital. Two radiologists with 10 years and 30 years of experience interpreted the CT images independently. All images were uploaded to the GE ADW 4.6 workstation, and the coronal and sagittal images were reconstructed using the Multi-Planar Reformation (MPR) technique with slice thickness of 3 mm and slice spacing of 3 mm. The degree of enhancement of the tumor was based on dynamic CT imaging using HU attenuation, in which “obvious enhancement” was defined as > 40 HU, “moderate enhancement” as > 20 HU and “mildly enhancement” as < 20 HU.

## Results

### Clinical manifestations

There were 21 GGT patients in total, with 6 males (28.6%) and 15 females (71.4%), a male to female ratio of nearly 1:2, and age range 42–64 (54.70 ± 7.27) years. Two of the patients had a history of *H. pylori* infection. Fourteen of the 20 benign cases had abdominal pain, two had blood in their stool, two had acid regurgitation and heartburn, and two were discovered during physical examination. The median time from symptom onset to diagnosis was 9 months (3–60 months), and none of them had a history of digestive diseases or a family genetic history. The detection of tumor markers was not increased in the benign cases. In this group, 16 cases were treated surgically, and 4 cases were treated with gastroscopic resection. Table 1 summarizes the clinical characteristics and CT features of glomus tumor, GIST, and heterotopic pancreas.

**Table 1:** Comparison between GGT and GST, heterotopic pancreas

	GGT	GST	Heterotopic pancreas	*P*_1_^a^	*P*_2_^b^
Location				0.000	0.321
Antrum	16 (80.0%)	5 (16.7%)	19 (63.3%)		
Body	4 (20.0%)	9 (30.0%)	9 (30.0%)		
Cardia and fundus	0 (0.0%)	16 (53.3%)	2 (6.7%)		
Main symptoms				0.721	0.119
Epigastric comfort/pain	16 (80.0%)	21 (70.0%)	29 (96.7%)		
Haematemesis/melena	2 (10.0%)	5 (16.7%)	0 (0.0%)		
Physical examination	2 (10.0%)	4 (13.3%)	1 (3.3%)		
Shape				0.009	0.001
Round	12 (60.0%)	12 (40.0%)	4 (13.3%)		
Quasi-round	8 (40.0%)	7 (23.3%)	20 (66.7%)		
Irregular shape	0 (0.0%)	11 (36.7%)	6 (30.0%)		
Growth pattern				0.032	0.004
Endophytic	8 (40.0%)	23 (76.7%)	25 (83.3%)		
Exophytic	4 (20.0%)	2 (6.7%)	3 (10.0%)		
Mixed	8 (40.0%)	5 (16.7%)	2 (6.7%)		
Ratio of long to short diameter	1.01 ± 0.15	1.18 ± 0.30	1.61 ± 0.84	0.02	0.003
Enhancement degree				0.000	0.001
Mildly enhancement	0 (0.0%)	4 (13.3%)	4 (13.3%)		
Moderate enhancement	0 (0.0%)	16 (53.3%)	11 (36.7%)		
Obvious enhancement	20 (100.0%)	10 (30.3%)	15 (50.0%)		
Enhancement pattern				0.005	0.287
Persistent enhancement	12 (60.0%)	24 (80.0%)	20 (66.7%)		
Continuous enhancement	2 (10.0%)	6 (20.0%)	6 (20.0%)		
Reduced enhancement	6 (30.0%)	0 (0.0%)	4 (13.3%)		
Enhancement pattern				0.166	0.630
Homogeneous	12 (60.0%)	12 (40.0%)	20 (66.7%)		
Heterogeneous	8 (40.0%)	18 (60.0%)	10 (33.3%)		
Ratio of CT value of lesion to abdominal aorta in arterial phase	0.41 ± 0.11	0.20 ± 0.05	0.24 ± 0.62	< 0.001	< 0.001
Ratio of CT value of lesion to abdominal aorta in venous phase	0.81 ± 0.20	0.48 ± 0.12	0.58 ± 0.14	< 0.001	< 0.001

^a^Comparison between GGT and GST

^b^Comparison between GGT and heterotopic pancreas

For the malignant case, a 63-year-old woman, the main complaint was upper abdominal pain and the time from symptom onset to diagnosis was 4 months. Among tumor markers, CA-125 (42.60 U/ml) was elevated. In this case, a distal gastrectomy was performed, as well as postoperative chemotherapy. There was no evidence of recurrence during the follow-up 16 months after the surgery.

### Pathological and other examinations

Twenty cases of GGT were found to be benign, with only one case being found to be malignant. Microscopically, tumor interspersed and grew between the muscularis propria of the gastric wall and contained many blood vessels, and one case had small focal calcification. The tumor boundary was distinct without capsule formation. The glomus cells were small, uniform, round, and showed perivascular hemangiopericytoma-like or solid nest-like structures without nuclear pleomorphism or mitotic figures. The tumor cells of the malignant case showed diffuse growth and high density. Obvious enlargement of nuclear volume, prominent cellular atypia, mitotic figures was observed (Fig. 2E, F, G), ulceration could be seen in the gastric mucosal surface. In 20 cases of benign GGT, tumor thrombus was found in 4 cases, nerve invasion in 8 cases, involvement of submucosa and mucosa in 6 cases. On immunohistochemical examination of the 20 benign cases, the tumor cells showed positive staining for SMA and Collagen-IV. CD34 was positive in 10 cases. Partial expression of Caldesmon or Calponin were moderately positive. Focal positivity for SYN was observed in 14 cases. Our group’s results for the remaining stains, such as CD56, CD117, CKPAN, DOG1, and S-100 proteins, were all negative in the 20 benign cases. The Ki-67 proliferation index of were 1–5% (mean 3%). Immunohistochemistry of the malignant case showed positive staining for SMA, and negative for CD117, DOG1, S-100, Caldesmon, Desmin, CD34. The Ki-67 proliferation index of the malignant case was 30% + . BRAF 600E mutation was not detected in the malignant case.

**Fig. 1:**
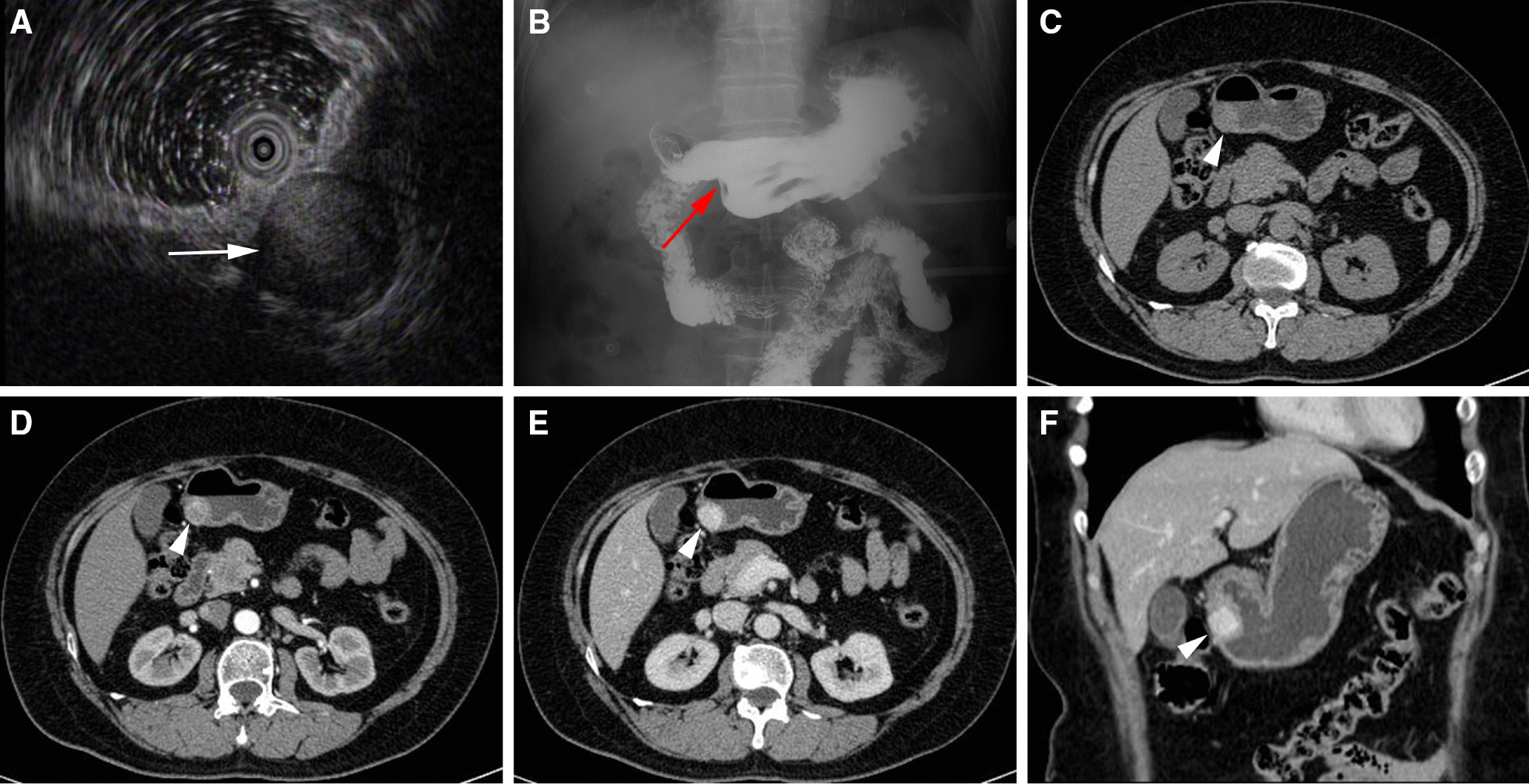
Images of benign GGT located on the greater curvature of gastric antrum. **A** Endoscopic ultrasonography shows hypoechoic lesion with local heterogenous echo (white arrow), which originates from muscularis propria. **B** Upper gastrointestinal barium meal radiography shows a round filling defect with smooth boundary in the greater curvature of gastric antrum (red arrow). **C** Non-contrasted CT shows round soft tissue density in the lesser curvature of the gastric antrum (arrowhead), the average CT value was about 41 Hu. **D** Contrasted CT shows obvious enhancement in arterial phase, the average CT value was about 92 Hu. **E** Portal venous phase image shows central filling enhancement, the average CT value was about 140 Hu. **F** The contrast enhancement coronal image in the portal phase shows homogenous density of the lesion with well-defined boundary

**Fig. 2:**
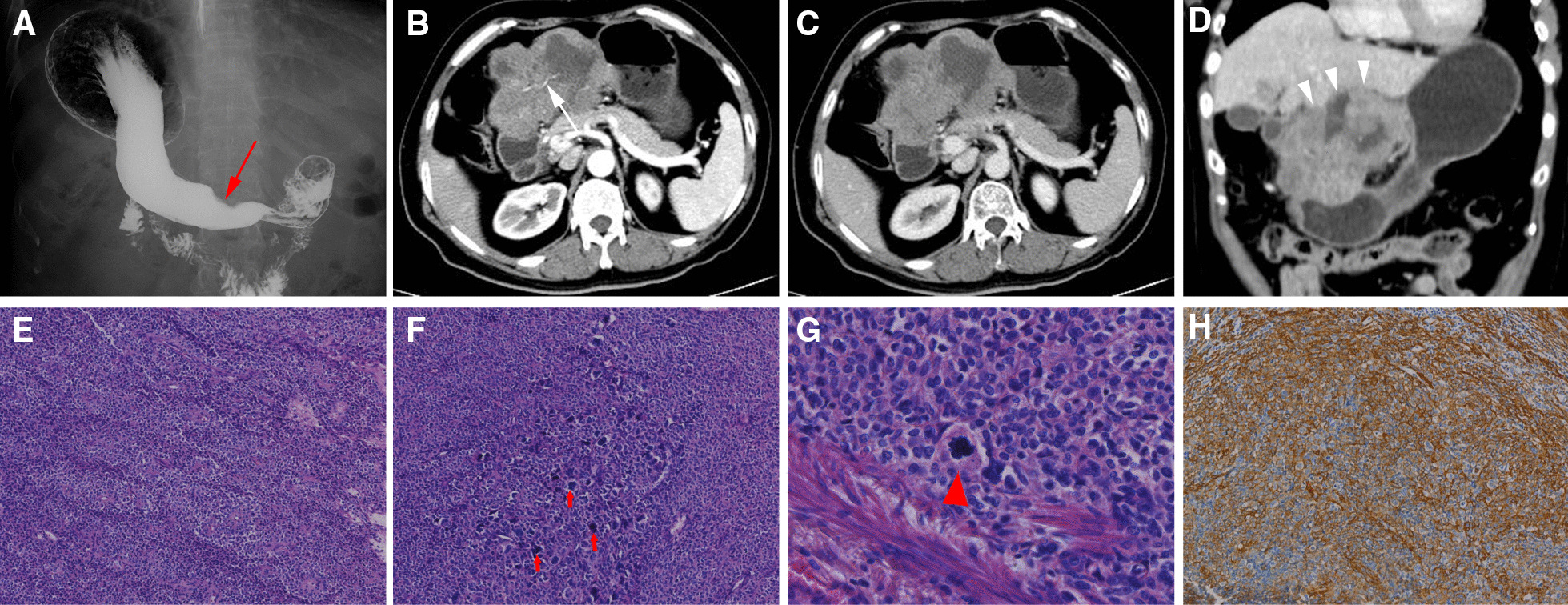
Images of malignant GGT of gastric antrum. **A** Upper gastrointestinal barium meal radiography shows a large filling defect in the lesser curvature of the stomach (red arrow). **B** Arterial phase image shows irregular mass of gastric antrum with deeply lobulated margin, thickening left gastric artery supplying blood can be seen (white arrow). **C** Portal venous phase image shows heterogeneous persistent moderate enhancement. **D** Coronal image of portal phase shows that the mass broke through the gastric wall and invade the liver with poorly defined boundary, cystic change and necrosis inside the tumor could be seen (arrowhead). **E** The round tumor cells with perivascular distribution were seen (HE,100 ×). **F** Prominent nucleoli and cellular atypia was seen in some areas (red arrow) (HE,100 ×). **G** A mitotic figure surrounded by neoplastic cells was seen (arrowhead) (HE,400 ×). **H** Tumor cells demonstrate the cytoplasmic staining with SMA (200 ×)

Endoscopic ultrasound was performed on 12 patients, 6 of whom were hypoechoic (Fig. 1A), 4 of whom were slightly hyperechoic, 2 of whom were hyperechoic, 6 of whom had homogeneous echo, and 6 of whom had inhomogeneous echo. Upper gastrointestinal radiography was performed in 6 cases, 5 of which revealed a quasi-circular filling defect with a smooth boundary (Fig. 1B), and 1 case revealed an irregular filling defect and a niche with an obscure boundary (Fig. 2A).

### CT findings

All the 21 patients were single lesion. Among the 20 benign cases, 4 were in the gastric body, 16 were in the gastric antrum. One malignant case was located at the gastric antrum. 20 benign cases were quasi-round or round protruding into the gastric cavity with well-defined boundary, with no ulcers on the surface or signs of invasion (Fig. 1C). Among the 20 benign GGTs, the ratio of long to short diameter was 1.01 ± 0.15, CT values of plain scan ranged from 27 to 54 (38.60 ± 8.21) Hu. In the arterial phase, all cases showed significant enhancement; 10 cases showed homogeneous enhancement, 10 cases showed heterogeneous enhancement (Fig. 1D), and 12 cases showed central filling enhancement (Fig. 1E, F). 12 patients showed persistent enhancement (the peak CT value occurred in the venous phase), 6 patients showed reduced enhancement (the peak CT value occurred in the arterial phase), and 2 patients showed continuous enhancement (CT value remained nearly the same toward arterial phase and venous phase). The ratio of CT value of lesion to abdominal aorta in arterial phase was (0.41 ± 0.11), and the ratio of CT value of lesion to abdominal aorta in venous phase was (0.81 ± 0.20). A malignant case with a long diameter of 150 mm and a thick diameter of 30 mm has an irregular boundary, penetrates the stomach wall, and invades the liver, with an unclear boundary and internal necrosis (Fig. 2D). The mass shows heterogeneous persistent moderate enhancement on the contrast CT images (Fig. 2C, D), with thickening left gastric artery supplying blood in the arterial phase (Fig. 2B).

## Discussion

GT is rare in stomach which accounts for approximately 1% of gastric mesenchymal tumors [4, 9] and was first reported by Kay et al. in 1951[10]. GGT was commonly found in the gastric antrum. Among the 21 cases in this study, 17 cases occurred in the gastric antrum, and 4 cases occurred in posterior wall of gastric body near gastric antrum, which were consistent with the literature report [11]. GGT is more common in middle-aged and elderly women with atypical clinical manifestations. Due to the lack of typical clinical and endoscopic characteristics, it’s highly needed to differentiate GGT from other richly vascularized tumors such as GST and heterotopic pancreas [12]. Some patients may have gastrointestinal symptoms such as dull abdominal pain, acid regurgitation and belching; however, only a few patients present as gastrointestinal bleeding or chronic anemia*.* In this group, 15 cases were middle-old females in this group, and the clinical symptoms of GGT were similar with other gastric submucosal tumors, with no statistical difference of clinical symptoms found when compared with GST and heterotopic pancreas respectively. The vast majority of GGT are benign, malignant GGT is extremely rare [13]*.* In our group, one case presenting with upper abdominal pain was confirmed as malignant GGT. The etiology and mechanism of GGT are still not clear, while a few cases are familial and may be related to 1p21-22 [11].

Glomus tumors are classified as vascular pericyte tumors in the WHO classification of soft tissue tumors. GT is classified into bulbar tumor, bulbar hemangioma, bulbar hemangiomyoma, and a few special types such as myxoid hemangioma, eosinophilic hemangioma, epithelioid hemangioma, and others based on the proportion of smooth muscle cells, globular cells, and vascular components. However, tissue classification is not related to the biological behavior of the tumor. Folpe et al. divided GT into benign tumor, malignant tumor or tumor with uncertain malignant potential [14]. The histopathological characteristics of benign GT were obvious proliferation of smooth muscle cell and tumor nest surrounded by glomus cells around numerous small capillary vessels. Glomus cells are mostly small, round, and uniform without nuclear atypia or mitotic figures. Occasionally, glomus cells will exhibit venous or nerve invasion. In our study, tumor thrombus was found in 4 cases, nerve invasion was found in 8 cases. These morphological characteristics are not indicators for judging the biological behavior of glomus tumor. In addition, the histopathological morphology of tumor in these cases was mild and the cell proliferation index was low, with no recurrence or metastasis occurred. We hypothesized that growth pattern associated with blood vessels and nerves of GGT may be a manifestation of local invasion of tumor tissue, which was different from the traditional vascular tumor thrombus and nerve invasion. Calcification can be seen in some cases [14]. Immunohistochemical stains is positive for MSA, SMA, Collagen IV, H-caldesmon, Calponin and Vimentin, and it is negative for CD-117, DOG1, desmin, chromogranin, S-100 protein and cytokeratin [15]. In our study, SMA and collage IV of the 20 benign cases were strongly expressed in all the tumor cells. Partial expression of Caldesmon or Calponin were moderately positive. Focal positivity for SYN was observed in 14 cases. CD34 was positive in 10 cases. These results were consistent with the literature report. The diagnosis of malignant GT includes three criteria: moderate to marked nuclear atypia together with mitotic activity of more than 5 mitoses per 50 high power fields, atypical mitotic figure or tumor size greater than 2 cm with deep location [14, 16]. The malignant glomus tumor should feature at least one of the first two previously mentioned criteria. In our study, we found prominent cellular atypia and mitotic figures in one case that was confirmed to be malignant GGT with a positive immunohistochemical staining for SMA.

GGTs are usually located in the submucosa and muscularis propria of the gastric wall. In our study, 12 patients had ultrasound gastroscopy, which revealed a low-echo or high-echo mass with homogeneous or inhomogeneous echo. Ultrasound gastroscopy can show the location of the tumor in the gastric wall, however, it is easily confused with other gastric submucosal tumors due to the lack of specificity. The upper gastrointestinal radiography showed round or quasi-circular filling defects with smooth boundary. Non-contrast CT images show single soft tissue lesion in the submucosa of gastric antrum with endophytic, exophytic or mixed growth. In our study, endophytic growth and mixed growth were more common. Most of GGTs are benign with smooth edge and well-defined boundary. Peri-gastric fat space is clear with no lymph node enlargement. Some literatures have reported spotty calcification in GGT due to the deposition of micro-phlebolith [17]. However, no fat or calcification was found on CT images in our group. CT can show smooth gastric mucosa covering the surface of the mass, suggesting that GGT originates from the submucosa. The degree of enhancement was consistent with the surrounding gastric mucosa. No mucosal ulcer was observed in the 20 benign cases which was small in size and the largest diameter of the lesions was less than 3 cm, which may be related to the slow growth of the tumor. Contrast enhanced CT images show obvious enhancement in arterial phase and persistent enhancement in venous phase. In our study, all the 20 benign cases showed obvious enhancement in arterial phase, of which 12 cases showed central filling enhancement, which was similar to the enhancement pattern of hemangioma corresponding to rich capillaries in pathology. 10 cases showed heterogeneous density on the contrast CT images, in which the area of low density was considered as cystic change caused by perivascular bleeding and degeneration [18].

Malignant GGT is extremely rare and has been reported only in a few literature [13–15, 19, 20]. Most of these papers were case reports about pathology, however, no article about the radiological feature of malignant GGT was published to date. The imaging characteristics of malignant GGT need to be further studied. Malignant GGT can invade the entire gastric wall with poor-defined boundary. Toti et al. [13] described a 72-year-old male patient with anemia who had a malignant GGT with liver metastases, in whom CT showed inhomogeneous mass which was 6 cm in diameter located in the greater curvature. There was no evidence of recurrence 3 and 6 months after the surgery. Alsahwan et al. [15] reported one malignant GGT in a-56-year-old male patient presenting with upper gastrointestinal bleeding. His CT images showed large lobulated mass at the greater curvature with no local invasion or distant metastasis. There was no evidence of recurrence 15 months after the resection of gastric mass. In our study, 1 case of malignant GGT mass showed deep ulcer with lobulation and nodular indentation on the surface, and the mass was large with exophytic growth invading the liver. The mass showed persistent moderate enhancement on contrast CT and the blood supply artery was from thickening left gastric artery. There was no evidence of recurrence 16 months after the surgery. Given the scarcity of reported malignant GGT, the CT findings of malignant GGT need to be further studied and summarized.

CT examination can provide a wealth of valuable information on the morphology, size, internal structure, growth pattern and blood supply of the lesion, while MPR can show the accurate location of the GGT. Due to lack of typical clinical and endoscopic characteristics, GGT needs to be differentiated from other similarly manifesting gastric submucosal tumors such as GST and heterotopic pancreas [21, 22]. GST usually occurs in the cardia and fundus of the stomach with exophytic growth [23]. In our study, GGT and GST showed significant differences in morphology, growth location, growth pattern, enhanced degree, enhanced pattern. GST was larger than 3 cm in size and showed persistent moderate enhancement on contrast CT images, and the ratio of CT value of lesion to abdominal aorta on contrast CT images was significantly lower than that of GGT. Heterotopic pancreas usually occurs in the gastric antrum, and most of them are quasi-circular masses with obvious enhancement on contrast CT images [24, 25]. In our study, GGT and heterotopic pancreas showed significant differences in morphology, growth location, growth pattern, enhanced degree, enhanced pattern, ratio of long to short diameter, ratio of CT value of lesion to abdominal aorta. There were no significant differences in the location or shape of GGT and heterotopic pancreas. Ratio of long to short diameter of heterotopic pancreas was higher than that of GGT. Because GGT is hyper-vascular tumor, it is not difficult to distinguish GGT from other submucosal tumors including gastric leiomyoma, gastric schwannoma, gastric neuroendocrine tumors. Gastric leiomyoma usually shows homogeneous density and mild to moderate enhancement. Gastric schwannoma usually occurs in conjunction with cystic degeneration and is characterized by mild to moderate enhancement. The common CT manifestations of gastric neuroendocrine tumors (staged G1, G2) are mild to moderate enhancement on contrast CT.

## Conclusions

GGT is rare and most commonly occurs in the gastric antrum of middle-aged and elderly women with atypical clinical manifestations, multiphase CT enhancement usually shows persistent obvious enhancement especially in arterial phase which provide important value for the diagnosis of GGT. The enhanced degree and pattern are based on its histological features. CT findings can help in the differential diagnosis of GGTs and other submucosal tumors of the stomach. The final and correct diagnosis, however, is dependent on the pathological and immunohistochemical results. It is critical to continuously report and collect newly diagnosed GGT in order to improve clinical and imaging understanding of GGT.

## Data Availability

The datasets used and/or analyzed during the current study are available from the corresponding author on reasonable request.

## Abbreviations

CT: Computed tomography
GGT: Gastric glomus tumor
GT: Glomus tumor
GST: Gastric stromal tumor
MPR: Multi-Planar Reformation
